# Draft Genome Sequence of a *Serratia marcescens* Strain Isolated from a Preterm Neonatal Blood Sepsis Patient at the Royal Infirmary, Edinburgh, Scotland, United Kingdom

**DOI:** 10.1128/genomeA.00908-14

**Published:** 2014-09-11

**Authors:** K. A. Kropp, A. Lucid, J. Carroll, V. Belgrudov, P. Walsh, B. Kelly, C. Smith, P. Dickinson, A. O’Driscoll, K. Templeton, P. Ghazal, R. D. Sleator

**Affiliations:** a Department of Biological Sciences, Cork Institute of Technology, Bishopstown, Cork, Ireland; b NSilico Lifescience Ltd., Bishopstown, Cork, Ireland; c Department of Computing, Cork Institute of Technology, Bishopstown, Cork, Ireland; d Division of Pathway Medicine, University of Edinburgh, Edinburgh, Scotland, United Kingdom; e Microbiological Diagnostic Unit, Royal Infirmary, University of Edinburgh, Edinburgh, Scotland, United Kingdom

## Abstract

Herein, we report the draft genome sequence for isolate ED-NGS-1015 of *Serratia marcescens*, cultivated from a blood sample obtained from a neonatal sepsis patient at the Royal Infirmary in Edinburgh, Scotland, United Kingdom.

## GENOME ANNOUNCEMENT

*Serratia marcescens* is a Gram-negative, rare, but clinically important nosocomial pathogen that causes meningitis and blood sepsis (1–3). Preterm neonates are a highly susceptible patient group for bacterial infections (4–6) and rapid detection of blood sepsis and the causative agent are critical first steps to enable proper treatment (7–9). The ClouDx-i project aims to extend our knowledge of currently circulating pathogenic strains linked with blood sepsis in neonates to inform the development of new molecular diagnostic assays. Herein, we present the draft genome of a *Serratia marcescens* strain isolated from a preterm neonate in Edinburgh in 2013. Positivity for blood sepsis and species identification were confirmed by classical microbiological techniques.

The isolate was grown overnight at 37°C on Luria broth (LB) agar, and genomic DNA was isolated using Qiagen genomic tips (Venlo, Limburg, Netherlands). Genomic DNA was fragmented (fragments 2 to 10 kb) using sonication and a non-size-selected genome library was produced using the Nextera mate pair kit (Illumina, San Diego, CA). This library was then sequenced on an Illumina MiSeq using MiSeq Reagent kit version 3. Genomic sequence assembly, analysis, and automated reporting were carried out using Simplicity (10). This approach produced 1,969,069 total reads, resulting in an average 117-fold coverage. The average G+C content was 59.91%. For sequence assembly, we used a *de novo* assembly pipeline based on the SPAdes version 3.10 assembly tool with k-mer sizes from K21, K33, K55, K77, K99 to K127, resulting in 166 contigs, of which 7 were >1,000 bp, representing 98.60% of the total sequence information, with the largest contig being 3,811,471 bp. Postassembly processing was performed using SPAdes, and only scaffolds of >1,000 bp were considered when estimating the genome length as 5,128,447 bp. We annotated the genome with Prokka (11) and used the identified 16S rRNA gene to confirm the species as *Serratia marcescens*. A scaffold of the genome was produced with Contiguator2, and we identified the closest related strain by BLASTing the scaffold, returning strains *S. marcescens* SM39 and FS14 as closely related but not identical, as was evidenced by a large inversion and numerous small insertions and deletions in the genome. The genome was then screened using GLIMMER3 (12) identifying 4,887 open reading frames (ORFs). The predicted ORFs were compared to the UniProt TrEMBL database (13) using BLASTp, mapping 3,885 of the ORFs to the database. To identify potential virulence factors, we compared the assembled genome to a local database built from the VFDB (14) and Victors databases with BLASTp. Using a 75% amino-acid sequence identity cutoff, while only considering alignments longer than 100 amino-acids, we identified 115 potential virulence factors.

Samples were handled in accordance with local ethical approval by the ethics committees of the NHS Lothian SAHSC Bioresource and NHS Research and Development Office, Project ID 2011/R/NE/01, and the HSS BioResource Request ID 13/ES/0126.

### Nucleotide sequence accession numbers.

This whole-genome shotgun project has been deposited at DDBJ/EMBL/GenBank under the accession number JPWM00000000. The version described in this paper is version JPWM01000000.
